# Subjective Well-Being and Active Life Expectancy in Japan: Evidence From a Longitudinal Study

**DOI:** 10.1093/geroni/igac075

**Published:** 2022-12-13

**Authors:** Yuka Minagawa, Yasuhiko Saito

**Affiliations:** Faculty of Liberal Arts, Sophia University, Tokyo, Japan; College of Economics, Nihon University, Tokyo, Japan

**Keywords:** Disability, Health expectancy, Life satisfaction, Mortality

## Abstract

**Background and Objectives:**

Existing research has suggested that older adults in Japan enjoy phenomenal physical health status, but they have poor subjective well-being (SWB). Limited empirical evidence exists, however, on how physical health and SWB intersect and are linked to the lives of older men and women in Japan. Using the concept of health expectancy, this study examines the role of SWB, as measured by life satisfaction, in the mortality and morbidity experiences of Japanese adults aged 65 years and older.

**Research Design and Methods:**

We used the nationally representative Nihon University Japanese Longitudinal Study of Aging, 1999–2009. Our measurement of morbidity is disability, based on difficulty in activities in daily living (ADLs) and instrumental ADLs. We use the Interpolation of Markov Chains approach to compute life expectancy (LE), LE without disability (active LE), and LE with differing severity of disability for those who are satisfied with life and for those who are not.

**Results:**

We documented significant differences in LE and active LE by the state of life satisfaction among older adults in Japan. Men and women who are satisfied with life are expected to live longer and spend more years without having disability compared to those who are not satisfied. We found no differences in the length of life with disability of differing severity by the state of life satisfaction.

**Discussion and Implications:**

Our results highlight the important role of SWB at older ages in Japan, because it is directly related to the physical health of its aging population. Fully understanding the health of the older population requires research that focuses on both objective and subjective dimensions of well-being.

**Translational Significance:** This study examined the role of subjective well-being (SWB), as measured by life satisfaction, in the health expectancy of older men and women in Japan. We documented significant differences in the length of active life by the state of life satisfaction, suggesting the importance of SWB in later life. This point is of particular importance in the years of the coronavirus disease 2019 pandemic, when older adults are at greater risk of being socially isolated.

## Background and Objectives

With the fast pace of population aging worldwide, there has been a growing interest in the quality of later life. The United Nations (UN) declared the period between 2021 and 2030 as the UN Decade of Healthy Ageing, the global initiative to promote the well-being of older adults, their families, and the communities in which they live (WHO, 2022). Quality of life is a multifaceted concept and includes both objective and subjective dimensions (Diener, 1984; Diener et al., 1999). The objective approach gives particular attention to the capabilities of individuals to have fulfilling lives, and it focuses on measurements, such as physical health status, educational attainment, employment status, and social participation. Conversely, the subjective approach, known as subjective well-being (SWB), is based on a person’s subjective evaluations of his or her life (D’Acci, 2011). Past research has identified three components of SWB: judgments about one’s life (e.g., overall life satisfaction), evaluations of specific life domains (e.g., satisfaction about family and work), and affective reactions (e.g., experiences of positive and negative emotions; Diener, 1984; Diener et al., 1999).

A large literature has investigated how SWB changes with age. Some researchers have found that SWB tends to be higher in later life (Blanchflower & Oswald, 2008; Gerstorf et al., 2007), while others reporting the opposite (Jivraj et al., 2014; Netuveli et al., 2006). There are several reasons to suspect that older people may have lower SWB. First, physical health status deteriorates with age. Compared to younger adults, older persons are likely to have worse self-rated health status (Andersen et al., 2007), mobility restrictions (Montgomery et al., 2020), and cognitive impairments (Crimmins et al., 2011). Physical health is a key determinant of SWB at advanced ages. Among older men and women in England, for instance, poor physical health conditions, namely disability, are related to lower SWB (Gerstorf et al., 2007). Second, older people receive limited social support, which in turn may negatively influence their health (Ejlskov et al., 2019). A loss of family members through death is a major health threat in later life. Previous studies have reported the adverse health consequences of widowhood, such as heightened risk of death (Hughes & Waite, 2009), hospitalization (Laditka & Laditka, 2003), and distress (Carr et al., 2001).

Japan offers an interesting case to investigate SWB at advanced ages for two key reasons. First, the Japanese population is aging at an extraordinary pace. According to the 2020 census, 28.6% of the population was older than 65, up from 26.7% in 2015 (Statistics Bureau, 2020). The share of the older population is expected to reach 38.4% in 2065 (Cabinet Office, 2020a). Continued increases in the number of older adults have important implications for Japanese social policy, especially when the first baby boomers, known as the *dankai* generation, reach age 75 in 2025. Another important feature includes the physical health status. In 2020, life expectancy (LE) at age 65 stood at 20.05 years for men (changing from 10.16 years in 1947) and 24.91 years for women (changing from 12.22 years in 1947; Statistics Bureau, 2020). Japanese adults also spend many years in good health, as evidenced in extensions in the length of life in good health (Yong & Saito, 2009).

Second, SWB remains relatively low among older individuals in Japan. The number of reported cases of abuse of older people has continued to increase. Major types of abuse by caregivers (excluding someone working at care facilities) include physical and emotional abuse, as well as neglect (Cabinet Office, 2020a). The experience of abuse yields unbeneficial health consequences, as in greater risk of disability (Schonfeld et al., 2006), institutionalization (Dong & Simon, 2013), and depression (Mouton et al., 2010). Additionally, social isolation in later life is a pressing social problem. Due to the demise of multigenerational households (Takagi & Silverstein, 2006), a growing number of older adults is now living and dying alone. In 2019, in the 23 districts of Tokyo, 3,936 persons aged 65 and older died alone, a phenomenon known as lonely deaths, and the number of lonely deaths increased by 80% between 2009 and 2019 (Cabinet Office, 2020a). Also, suicide rates among older adults remain high. In 2020, 8,126 people aged 65 and older committed suicide, an increase from the 7,953 suicides in the previous year (Cabinet Office, 2020a). Such an increase may be an indication of the adverse consequences of coronavirus disease 2019 (COVID-19) for the lives of older people.

Furthermore, Japanese people have relatively poor SWB when compared with international standards. A repeated cross-sectional survey conducted in Japan, Germany, Sweden, and the United States shows that Japanese adults aged 60 and older are less satisfied with life compared to their western counterparts. According to the 2020 survey results (Cabinet Office, 2020b), 81.5% of Japanese respondents answered that they were satisfied with life (“satisfied” and “somewhat satisfied,” combined), a much lower percentage than percentages in the United States (94.6%), Sweden (92.2%), and Germany (91.6%). Men had lower levels of life satisfaction (78.6%) than women (84.2%). Also, results point to downward trends in SWB: the percentage of respondents who were satisfied with life decreased from 84.2% in 2015.

While older adults in Japan enjoy phenomenal physical health status but have poor SWB, several important issues remain unanswered. First, the relationship between objective and subjective dimensions of well-being remains to be established. This is because the literature on Japanese people’s objective and subjective well-being remains largely separate, and thus relatively little is known how SWB may be related to physical health status among older adults. A small body of work that does focus on the SWB and health of the Japanese population has examined mortality and health separately (Willroth et al., 2020). With recent increases in chronic illnesses, it has become critical to jointly assess mortality and morbidity, because longer lives do not necessarily mean healthy lives (Crimmins et al., 1994). Second, there is a large volume of work on health expectancy among the Japanese population, but most of existing research is based on the prevalence of a specific health condition, taken from cross-sectional survey data. The period prevalence-rate life table approach computes the expected duration of life for a member of a life table cohort given the current age-specific prevalence rates of morbidity and mortality. It is important to note that individuals experience changes in health status, such as developing a health problem, recovering from it, or dying from it (Crimmins et al., 2009; Jagger et al., 2007).

Using the concept of health expectancy that incorporates mortality and morbidity, the present study investigates the role of SWB among Japanese adults aged 65 and older. Our measurement of morbidity is disability, and we focus on life satisfaction as a measurement of SWB. We choose to focus on life satisfaction, because prior research has used it to examine the relationship between SWB and physical health status (Lutz et al., 2021; Solé-Auró et al., 2018). There are two main objectives. First, we examine if LE and health expectancy vary by the state of life satisfaction. More specifically, we estimate LE, LE without disability (active LE, hereafter), and LE with disability of differing severity for those who are satisfied with life and for those who are not and then explore differences between these two groups. Second, we employ the multistate life table approach to compute incidence-based health expectancy. In contrast to the prevalence-based method, this approach accounts for the impacts of the changing prevalence of morbidity and produces estimates of incidence-based health expectancy (Crimmins et al., 2009). With this research, we contribute to the literature by (a) quantifying the role of life satisfaction in the form of health expectancy, and (b) offering evidence from Japan, a country where older people enjoy good physical health status, yet their SWB remains relatively low.

## Research Design and Methods

### Data

The Nihon University Japanese Longitudinal Study of Aging (NUJLSOA) was used for the analysis. We used the data from the baseline survey of 1999 and four longitudinal follow-ups in 2001, 2003, 2006, and 2009. The NUJLSOA is based on multistage probability sampling and provides a nationally representative sample of noninstitutionalized individuals aged 65 and older in Japan at baseline. The data were collected through in-person interviews based on a standardized questionnaire that covers a range of topics about the lives of older people, such as demographic and socioeconomic characteristics, physical, mental, and oral health conditions, health care utilization, health lifestyles, interpersonal relationships, and social inclusion. The response rate of the baseline survey was 74.6% and remained higher than 80% throughout follow-up interviews. This study utilized the baseline sample of 4,994 people (2,882 women and 2,112 men) who were followed through 2009. More details about the NUJLSOA survey have been published previously (Yong & Saito, 2012).

### Measures

Estimating health expectancy requires data on mortality and morbidity. In the NUJLSOA, death of a participant was reported by a family member through longitudinal follow-ups. There are in total 1,434 reported deaths between 1999 and 2009.

The key indicator of physical health is disability, measured by difficulty in activities in daily living (ADLs) and instrumental ADLs (IADLs). ADLs in this study comprise bathing, dressing, eating, getting out of a bed, moving around the home, toileting, and walking, and IADLs refer to preparing meals, using the telephone, managing money, shopping for groceries, doing light housework, taking medications, and using public transportation. Response categories include “difficult” and “not difficult.” Using the information on ADLs and IADLs, the following three types of disability states were defined: without disability (no difficulty in ADLs and IADLs), IADL disability (difficulty in performing one or more IADLs but no difficulty in ADLs), and ADL disability (difficulty in performing one or more ADLs). Active LE in this study denotes the average length of life without difficulty in ADLs and IADLs. Prior research has used the same definition of LE with disability of differing severity (Crimmins et al., 2009).

Our measurement of SWB is life satisfaction. While SWB can be measured by a range of variables, such as happiness, positive emotions, and meaning in life (Diener, 1984; Diener et al., 1999), this study focuses on life satisfaction as an indicator of SWB. Past research has suggested that life satisfaction is more reflective and less volatile than happiness (Lutz et al., 2021), and it has been used to assess the relationship between SWB and physical health status (Solé-Auró et al., 2018). In the NUJLSOA, participants were asked whether they are satisfied with their life as a whole these days. Responses are given as “yes” or “no.” Individuals were considered as satisfied with life if they answered “yes” to the question.

### Analytical Strategy

We use the multistate life table approach to compute incidence-based health expectancy in the Interpolation of Markov Chains (IMaCh) computer program version 0.99r24 (Lievre et al., 2003). We define in total 12 transitions across three states of disability and death (Supplementary Figure 1). The IMaCh program estimates multinomial logistic regression models and calculates the annual rates of age-specific transitions across these states. These transition rates are then used as the input into a multistate life table to compute (a) the remaining years of life at a given age (i.e., LE), (b) LE without disability (i.e., active LE), (c) LE with IADL disability, and (d) LE with ADL disability. The data in the NUJLSOA were collected every 2–3 years, but the IMaCh program handles differences in the length of the intervals between each interview. Also, prior research has used the same analytical technique to compute health expectancy for older populations (Crimmins et al., 2009; Jagger et al., 2007), including those in Japan (Yong & Saito, 2012).

We then tested the role of life satisfaction in health expectancy measures. More specifically, we estimate LE, ALE, LE with IADL disability, and LE with ADL disability for those who are satisfied with life and for those who are not. The 95% confidence intervals, generated by the IMaCh program, allow us to detect significant differences, if any, in health expectancy by the state of life satisfaction. Life satisfaction is measured at each wave and included in the models as a time-varying covariate. We estimated life satisfaction-specific health expectancy separately for men and women.

## Results

Table 1 presents the summary of transitions, in terms of disability (i.e., without disability, with IADL disability, with ADL disability, and dead) and Table 2 presents the summary of life satisfaction (i.e., satisfied and not satisfied), measured at the beginning and at each consecutive survey wave for the period between 1999 and 2009. Most of the transitions occurred within the same disability state, except those with IADL disability at the beginning of a wave. In this group, the transition from IADL disability to ADL disability is the most common pattern of change (35.48% of all transitions). Individuals with ADL disability at the beginning of each wave had the largest number of transitions to death. Also, results in Table 2 show that, among those who were satisfied with life at the beginning of each wave, 84.38% of the transitions were classified as remaining satisfied, followed by 9.78% classified as dead, and 5.85% classified as not satisfied. In contrast, the change from not satisfied to satisfied was the most common pattern of transition in the not satisfied group, indicating positive changes and developments as individuals live into old age (Tornstam, 1996). The transition to death was more frequently observed for the state of not satisfied.

**Table 1.** Summary of Transitions Across the States of Health (%) Between 1999 and 2009

Initial status	Ending status				Total
	Without disability	With IADL disability	With ADL disability	Dead	
Without disability	79.67	9.20	5.43	5.70	100.00
With IADL disability	25.45	24.08	35.48	14.99	100.00
With ADL disability	12.60	5.91	51.77	29.72	100.00

*Notes*: ADLs = activities of daily living; IADLs = instrumental activities of daily living.

**Table 2.** Summary of Transitions Across the States of Life Satisfaction (%) Between 1999 and 2009

State of life satisfaction	Satisfied	Not satisfied	Dead	Total
Satisfied	84.38	5.85	9.78	100.00
Not satisfied	54.42	30.53	15.04	100.00

Next, we use the age-specific transition probabilities estimated above as the input into a life table and calculate LE, active LE, LE with IADL disability, and LE with ADL disability for those who are satisfied with life and for those who are not. Table 3 presents these estimates at age 65, in addition to the percentage of disability-free life. Several points are noteworthy. First, for men and women, there are significant differences in LE at age 65 by the levels of life satisfaction. Among men, there is a 3.96-year difference in LE at age 65 between those who are satisfied with life (21.34 years) and those who are not (17.38 years). The difference in LE at age 65 is slightly larger for women. There is a 4.49-year difference in female LE at age 65, ranging from 20.92 years among women who are not satisfied to 25.41 years among those who are satisfied. These differences in LE at age 65 are statistically significant for both genders.

**Table 3.** LE, LE Without Disability, LE With IADL Disability, LE With ADL Disability, and Percentage of Life Without Disability at Age 65, Stratified by the Level of Life Satisfaction

	Expected years of life (LE)	Expected years of life:			% of life without disability
		Without disability	With IADL disability	With ADL disability	
Men					
Satisfied	21.34 (19.85–22.26)	17.17 (16.52–17.82)	1.06 (0.84–1.28)	3.11 (2.59–3.63)	80.46 (77.41–83.51)
Not satisfied	17.38 (16.46–18.30)	13.03 (12.22–13.84)	1.16 (0.95–1.37)	3.19 (2.72–3.66)	74.97 (70.32–79.62)
Women					
Satisfied	25.41 (24.28–28.64)	17.64 (17.10–18.18)	1.84 (1.56–2.12)	5.93 (5.11–6.75)	69.41 (67.28–71.54)
Not satisfied	20.92 (19.96–21.88)	14.20 (13.48–14.91)	1.53 (1.30–1.75)	5.19 (4.65–5.74)	67.88 (64.46–71.30)

*Notes*: ADLs = activities of daily living; IADLs = instrumental activities of daily living; LE = life expectancy. The 95% confidence intervals are in parentheses.

Second, there are clear differences in active LE at age 65 by the state of life satisfaction. For men and women, the average length of life without disability at age 65 is significantly longer for those who are satisfied with life than those who are not. Active LE at age 65 was 17.17 years for men who are satisfied with life, while the result for those who are not satisfied was 13.03 years. Similar patterns are observed among women. At age 65, women who were satisfied with life could expect to live 17.64 years without having disability, while the result for their unsatisfied counterparts was 14.20 years. When these results are converted into the percentage of disability-free life (active LE divided by LE multiplied by 100), men and women who are satisfied with life are expected to spend greater proportions of their life without disability. Such differences by the state of life satisfaction, however, are not statistically significant.

Third, the length of life with disability of differing severity does not depend on the state of life satisfaction. For men and women, there are no significant differences in LE with IADL disability and LE with ADL disability by the level of life satisfaction. Men who are satisfied with life are more advantaged in terms of LE with IADLs and ADLs than those who are not, but differences between these two groups are not significant. The patterns are reversed among women, because those who are satisfied with life are expected to spend more years with IADLs and ADL disability compared to those who are not satisfied.

Estimates of LE, active LE, and LE with differing severity of disability at ages 75 and 85 are presented in Supplementary Tables 1 and 2. We found similar patterns: at ages 75 and 85, LE and active LE are significantly longer for men and women who are satisfied with life than those who are not. Importantly, there are no differences in terms of LE with IADL disability and with ADL disability.

## Discussion and Implications

Using the nationally representative sample on older adults in Japan, this study examined whether and to what extent LE and health expectancy depend on the state of life satisfaction. Our investigation yields the following findings. First, we noted substantial differences in LE and active LE by the state of life satisfaction. Men and women who were satisfied with life were expected to enjoy longer lives and spend more years without having disability compared to those who were not satisfied. These patterns are consistent across different ages. Yet, when results were interpreted in relative terms, the gap in the percentage of active life, between those who were satisfied with life and those who were not, was no longer significant. Second, there were no differences in the length of life with disability of differing severity by the state of life satisfaction. LE with IADL disability and ADL disability did not largely vary between those who were satisfied with life and those who were not.

Overall, these results suggest the important role of SWB, as measured by life satisfaction, in LE and active LE among older adults in Japan. This study combined life satisfaction, mortality, and morbidity in the concept of health expectancy and documented marked variations in LE and active LE by the levels of life satisfaction. Most of the existing research on health expectancy among the Japanese population has typically focused only the negative dimension of health, namely the presence of disability (Hashimoto et al., 2010). This is perhaps because poor health yields serious consequences to both individuals and society (Fredrickson, 2004). Our study departs from this traditional “negative health” approach and gives particular attention to variations in health expectancy by the levels of life satisfaction among older people in Japan.

The findings also imply the need for improving the quality of later life among older adults in Japan. Recent increases in abuse of older people and suicide rates are emerging evidence of poor SWB among older adults in the country. Active social involvement at advanced ages has well-documented health benefits, namely lowered mortality (Musick et al., 1999) and increased mental health (Li & Ferraro, 2005). Older people stand to benefit from active social engagement, as it provides them with a sense of purpose in life and fulfills the loss of roles after retirement (Ejlskov et al., 2019). In Japan, post-retirement employment has favorable effects on the well-being of older people (Weiss et al., 2005). Consistent with these past studies, the present findings confirm the length-of-life and life-free-from-disability health benefits of having higher levels of SWB in later life.

This study has several limitations. The first limitation concerns the measurement of SWB. The present study focused on life satisfaction, but there are multiple ways to conceptualize SWB. For example, happiness, based on a global judgment of one’s life as a whole, is an important aspect of SWB (Veenhoven, 1996). The NUJLSOA survey includes a question about happiness, but it is asked as a part of the Center for Epidemiological Studies—Depression scale. Respondents were thus asked about the frequency of feeling happy in the past week, rather than about rating their overall happiness. We nevertheless conducted analyses with the information on happiness and came to similar conclusions as the current study: Happiness is related to longer LE and active LE for men and women. In addition, our measurement of life satisfaction is based only on a dichotomous variable of “yes (satisfied)” and “no (not satisfied).” Life satisfaction can be measured through a wider range based on the Likert scale from 0 to 10, allowing for the investigation into subtle variations in the levels of life satisfaction (Lutz et al., 2021). Overall, future research will benefit from methodological considerations, including how to conceptualize and more precisely measure SWB.

The second issue is about the measurement of health. This study focused on disability, with particular attention given to the severity of disability. While we documented significant associations of life satisfaction with total LE and active LE, there were no variations in LE with disability of differing severity by the state of life satisfaction. These results are intriguing, but one should note that the relationship between SWB and health expectancy may depend on the definition of disability. One important area of future research will be to consider how to define disability and how SWB might translate into varying experiences of disability among older individuals.

The third issue has to do with the data. The NUJLSOA only includes community-dwelling adults at baseline. The omission of institutionalized individuals may create a discrepancy between our estimates and the actual health status of older people. Past evidence from the United States shows that incorporating the data on the institutionalized population could produce more accurate estimates of health in a survey (Crimmins et al., 2009). Using proxy respondents during follow-up interviews, the NUJLSOA collected information on the health status of those who moved into institutions, but proxies were not allowed for subjective information, including life satisfaction. One should note the possibility that the current estimates of LE and health expectancy measures may be overestimated, as we failed to capture the mortality and morbidity experiences of those in institutions.

Despite these limitations, this study presents an important first attempt toward understanding the relationship between objective and subjective dimensions of well-being among older people in Japan. The policy implications of the present analyses include the importance of designing welfare programs targeted at enhancing the quality of later life. Promoting the well-being of older persons is important and becoming even more so during the COVID-19 outbreak. The older population has been vulnerable during this pandemic, not only because they are likely to have preexisting health conditions, but also because the pandemic has drastically diminished opportunities for social involvement, putting this population at heightened risk of isolation (Noguchi et al., 2021). In Japan and elsewhere, more policy and research attention should be given to the subjective dimension of well-being among older adults, because it has a direct bearing on their physical health status.

## Supplementary Material

igac075_suppl_Supplementary_Material
