# Supplementary material for: Subjective Well-Being and Active Life Expectancy in Japan: Evidence From a Longitudinal Study
Source: Innov Aging. 2022 Dec 13;7(1):igac075. doi: 10.1093/geroni/igac075 (PMC9929276; doi:10.1093/geroni/igac075)
Supplement: igac075_suppl_Supplementary_Material [file igac075_suppl_supplementary_material.docx]

**Supplementary Table 1**. Life Expectancy, LE without Disability, LE with IADL Disability, LE with ADL Disability, and Percentage of Life without Disability at Age 75, Stratified by the Level of Life Satisfaction

|  | Expected years of life (LE) | Expected years of life: | | | % of life without disability |
| --- | --- | --- | --- | --- | --- |
|  |  | without disability | with IADL disability | with ADL disability |  |
| *Men* |  |  |  |  |  |
| Satisfied | 13.67 | 9.52 | 1.04 | 3.11 | 69.67 |
|  | (12.17-14.57) | (8.94-10.11) | (0.79-1.29) | (2.53-3.68) | (65.38-73.96) |
| Not satisfied | 10.36 | 6.25 | 1.08 | 3.04 | 60.30 |
|  | (9.72-11.00) | (5.71-6.78) | (0.88-1.27) | (2.63-3.45) | (55.15-65.44) |
| *Women* |  |  |  |  |  |
| Satisfied | 17.06 | 9.32 | 1.81 | 5.92 | 54.64 |
|  | (15.96-18.16) | (8.86-9.78) | (1.51-2.12) | (5.06-6.79) | (51.96-57.31) |
| Not satisfied | 13.40 | 6.70 | 1.50 | 5.20 | 49.99 |
|  | (12.72-14.08) | (6.22-7.18) | (1.28-1.73) | (4.69-5.71) | (46.62-53.56) |

Note. LE = Life expectancy; ADLs = activities of daily living; IADLs = instrumental activities of daily living. The 95% confidence intervals are in parentheses.

**Supplementary Table 2**. Life Expectancy, LE without Disability, LE with IADL Disability, LE with ADL Disability, and Percentage of Life without Disability at Age 85, Stratified by the Level of Life Satisfaction

|  | Expected years of life (LE) | Expected years of life: | | | % of life without disability |
| --- | --- | --- | --- | --- | --- |
|  |  | without disability | with IADL disability | with ADL disability |  |
| *Men* |  |  |  |  |  |
| Satisfied | 7.79 | 3.95 | 0.93 | 2.91 | 50.71 |
|  | (6.31-8.72) | (3.38-4.52) | (0.62-1.24) | (2.21-3.60) | (40.41-61.02) |
| Not satisfied | 5.52 | 2.07 | 0.86 | 2.59 | 37.50 |
|  | (4.88-6.16) | (1.54-2.60) | (0.66-1.05) | (2.18-3.00) | (27.84-47.17) |
| *Women* |  |  |  |  |  |
| Satisfied | 9.98 | 3.13 | 1.51 | 5.34 | 31.39 |
|  | (9.31-10.65) | (2.85-3.42) | (1.28-1.74) | (4.76-5.92) | (28.52-34.26) |
| Not satisfied | 7.55 | 1.82 | 1.17 | 4.55 | 24.16 |
|  | (6.87-8.22) | (1.34-2.30) | (0.95-1.40) | (4.04-5.06) | (17.82-30.50) |

Note. LE = Life expectancy; ADLs = activities of daily living; IADLs = instrumental activities of daily living. The 95% confidence intervals are in parentheses.


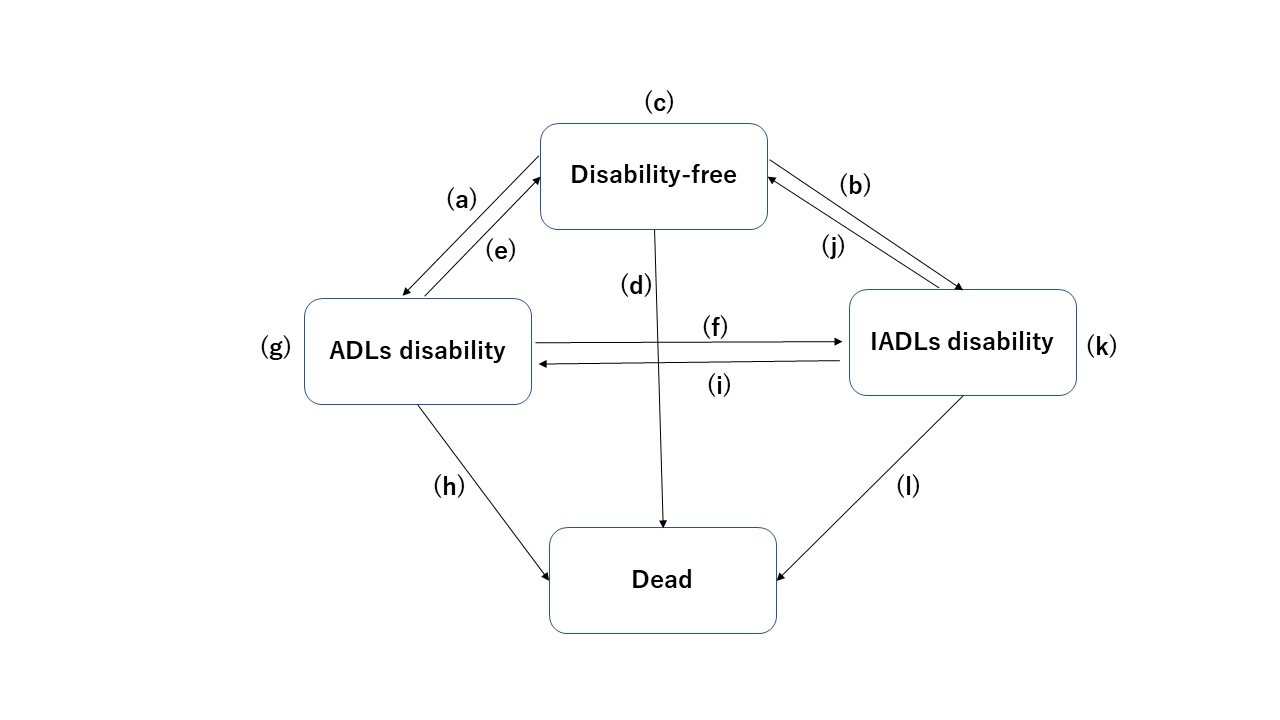


**Supplementary Figure 1.** Transitions across health states. (a) Moving from disability-free to activities of daily living (ADLs) disability, (b) Moving from disability-free to instrumental ADLs (IADLs) disability, (c) Remaining disability-free, (d) Death from disability-free, (e) Moving from ADL disability to disability-free, (f) Moving from ADL disability to IADL disability, (g) Remaining at ADL disability, (h) Death from ADL disability, (i) Moving from IADL disability to ADL disability, (j) Moving from IADL disability to disability free, (k) Remaining at IADL disability, (l) Death from IADL disability.
